# Identification of the Ideal Reference Site and Pain Threshold Values for the Placement of Electric Pulp Testers in Permanent Upper and Lower Anterior Teeth: A Cross-Sectional Study

**DOI:** 10.7759/cureus.58156

**Published:** 2024-04-12

**Authors:** Kanamarlapudi V Saikiran, Deepa Gurunathan, Esha Gayathri, Sivakumar Nuvvula

**Affiliations:** 1 Department of Pediatric and Preventive Dentistry, Saveetha Dental College and Hospitals, Saveetha Institute of Medical and Technical Sciences, Saveetha University, Chennai, IND

**Keywords:** electric pulp test, pulp sensible test, reference sites, pain threshold value, memojis pain scale

## Abstract

Introduction: Electric pulp testers (EPTs) are widely used diagnostic tools for diagnosing traumatized teeth. Several factors can affect the result of an electric pulp test. One such factor that will affect the diagnosis is the electrode tip placement. Hence, the current study aims to identify the most painful site and response time threshold in healthy anterior teeth.

Methods: A total of 90 individuals, 48 male and 42 female, aged 19 to 25 years, were recruited. An EPT was placed on three different sites: the cervical, middle, and incisal third of the labial surface of both upper and lower anterior teeth (central incisor, lateral incisor, and canine) with an appropriate electrolyte as a conducting medium. Later, the threshold values were recorded, and pain assessment was done using the Memojis pain scale (MPS). Finally, the data was analyzed statistically using the Mann-Whitney U test.

Results: Mean and standard deviation values of reaction time were collected from 540 EPT readings (three sites from 180 teeth). Among the three sites tested, the difference between the upper and lower central incisors was statistically insignificant (p > 0.05). Similarly, when upper and lower lateral incisors and canines were compared, a statistically significant difference was observed among the three sites (p<0.05). There was a significant difference (p<0.05) in the pain scores only on the incisal and cervical thirds of the upper and lower central incisors. Only the incisal third showed a statistically significant difference (p<0.05) between the pain scores in the upper and lateral incisors. At the same time, a statistically significant difference in the pain scores was observed among the three tested sites between the upper and lower canines (p<0.05).

Conclusion: Lower threshold values were appreciated at the incisal third of all the upper and lower anterior teeth for placing the EPT. Most individuals have experienced a score of 2 (hurts little bit) for the perceived pain using EPTs for both the upper and lower anterior teeth.

## Introduction

Traumatic dental injuries can cause reduced function and aesthetics by injuring the pulpal and periodontal structures as well as hard dental tissues. Following acute dental trauma, within the healing period, it is imperative to establish the exact pulpal condition of the affected teeth to restore both function and aesthetics effectively. However, this diagnosis becomes a challenge for the clinician due to transient loss of pulpal sensibility after trauma [1]. Various conventional methods are reported in the literature to determine pulpal vitality, such as CO_2_ snow, ice sticks, refrigerant sprays, ethyl chloride, heated gutta-percha, dual-wavelength spectrophotometry, laser doppler flowmetry (LDF), and pulse oximetry [2].

The electric pulp tester (EPT) is one such instrument that has been considered a standard clinical pulp diagnostic test. Magitot suggested the utilization of electrical energy in the field of dentistry, but Marshall first employed it in 1891 to assess pulp vitality [3,4]. The EPT elicits a pulpal response using an electric current. Its main objective is to assess the sensitivity of dental pulp, specifically the sensitivity of A delta fibers [5]. The EPT primarily evaluates the sensitivity of the pupal nerve without providing an assessment of the vascular condition of the pulp. The EPT can evoke sensations such as tingling, warmth, or pain, indicating the presence of vital pulp tissue.

Conversely, a lack of reaction to the EPT stimulus suggests indirectly that pulp may be necrosed or nonvital. Therefore, the EPT is not the true determinant of pulp vitality, as it only offers information on the innervations of the dental pulp and not from the vascular supply [6]. Certain studies have found that the EPT has high specificity and varying sensitivity. It has been widely accepted that the EPT is unreliable for immature teeth, recently traumatized teeth, or orthodontically treated teeth. In addition, the EPT is more reliable in identifying healthy teeth than teeth with diseased pulp tissues [5]. Multiple variables can influence the outcome of the electric pulp test, such as the thickness of enamel and dentin, the extent of sensory fibers (specifically A delta fibers), the orientation of dentinal tubules while the electrode tip is placed, the size of the pulp chamber, the presence of sclerotic dentin, the decrease in neural elements associated with aging, apprehension regarding electric shock, traumatic injuries, and immature tooth/teeth. Consequently, the presence of healthy pulp tissue may be misinterpreted as aberrant pulp tissue and vice versa [6].

The selection of the optimal position for placing the EPT tip carries significant clinical significance. Precise positioning can guarantee accurate measurement and reduce the risk of false-positive results during procedures [7,8]. It is crucial for clinicians to carefully consider factors such as patient anatomy and the specific procedure being performed when determining the ideal location for the EPT tip. Therefore, the present study aimed to determine the ideal location and level of pain that people experienced after placing EPT tips on both the maxillary and mandibular anterior teeth at three different locations.

## Materials and methods

Study setting

The present study was conducted in the Department of Paediatric and Preventive Dentistry. The study received ethical approval from the institutional ethics committee (IHEC/SDC/FACULTY/23/PEDO/262), and the study was conducted from June 2023 to August 2023.

Participant selection

The estimation of the sample size was done based on the previous study by Tian et al. [5] A total of 90 individuals, 48 men and 42 women, aged 19 to 25 years, were enrolled in the study based on the following inclusion and exclusion criteria: teeth free of dental caries, teeth free from restorations and root canal treatment, and teeth free from visible cracks. Individuals who underwent periodontal and orthodontic treatment, without signs or symptoms of pulp inflammation, such as the presence of pain, tenderness on the percussion, mobility, or existence of a sinus tract; individuals with a recent history of dental trauma; and individuals with any history of narcotics, alcohol, or nonsteroidal anti-inflammatory drugs before the procedure were excluded from the study.

Procedure

Written informed consent was obtained from all the participants after a thorough explanation and understanding of the study process. After meeting the inclusion criteria, 540 surfaces on anterior teeth were selected, including the central incisor, lateral incisor, and canine from the maxillary and mandibular arches. Later, all included teeth were carefully isolated with cotton rolls and subsequently dried using a gauze piece by one of the experienced pediatric dentists. According to the manufacturer's instructions, electric pulp testing was performed using a Waldent electric pulp tester (Waldent Innovations Pvt Ltd, New Delhi, India) with a range of 0-40 (vital teeth). The rate of increase was slowly adjusted to permit reliable determination of the first perception of the stimulus; the measuring scale ranges from 0 to 40 units. Following isolation, toothpaste (Colgate, Colgate Palmolive India Ltd., Gujarat, India) was used to coat the electrode tip of the EPT, which served as a conducting medium. The electrode tip was then placed on the surface to be tested. The participant was instructed to use his thumb and forefinger to hold the EPT lip clip to complete the circuit. Participants were advised to raise their hands as soon as they felt any warmth, stinging, tingling, or pain, and the pulp tester's digital display readout at that time was recorded. An initial test was conducted on a tooth not included in the study to accurately identify the sensation caused by an electrical impulse.

**Figure 1:**
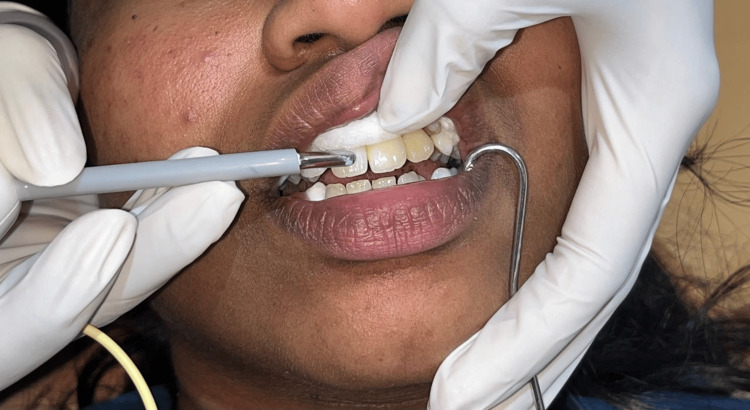
EPT tip placement on maxillary central incisor Image credit: Deepa Gurunathan EPT: Electric pulp tester

**Figure 2:**
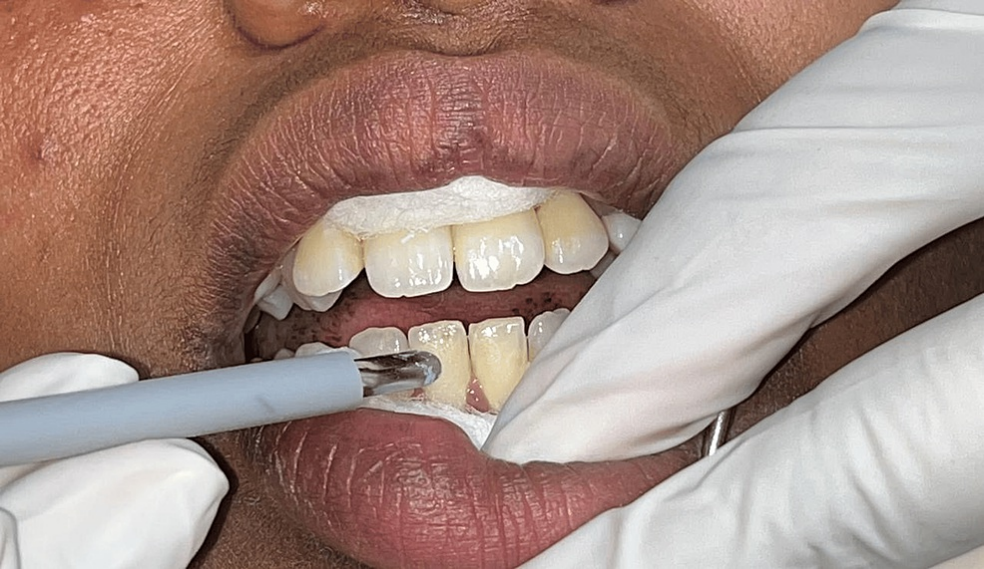
EPT tip placement on mandibular central incisor Image credit: Deepa Gurunathan EPT: Electric pulp tester

The cervical, middle, and incisal thirds of the labial surface were examined in the anterior maxillary and mandibular teeth of the labial surface (Figures 1, 2). Each participant was guaranteed a recovery interval of at least two minutes between tests to allow pulp tissue to return to its normal state, preventing the appearance of nerve accommodation and maintaining the regularity of the test sequence.

Outcomes and assessment tool

The present study recorded the pulp vitality score on EPT, pain level, and time taken for EPT response for the corresponding tooth surface. On the EPT test, the tooth pulp was considered vital if the patient felt mild pain or a warm, stinging, tingling, or uncomfortable sensation. Furthermore, in those cases where the patient felt pain, the pain was recorded using the Memojis Pain Scale (MPS) (score 0 represents no hurt, score 2: hurts a little bit, score 4: hurts a little more, score 6: hurts even more, score 8: hurts a whole lot, score 10: hurts worst). Simultaneously, the time duration between the start of the EPT application and the feeling of pain or tingling sensation was recorded.

**Figure 3:**
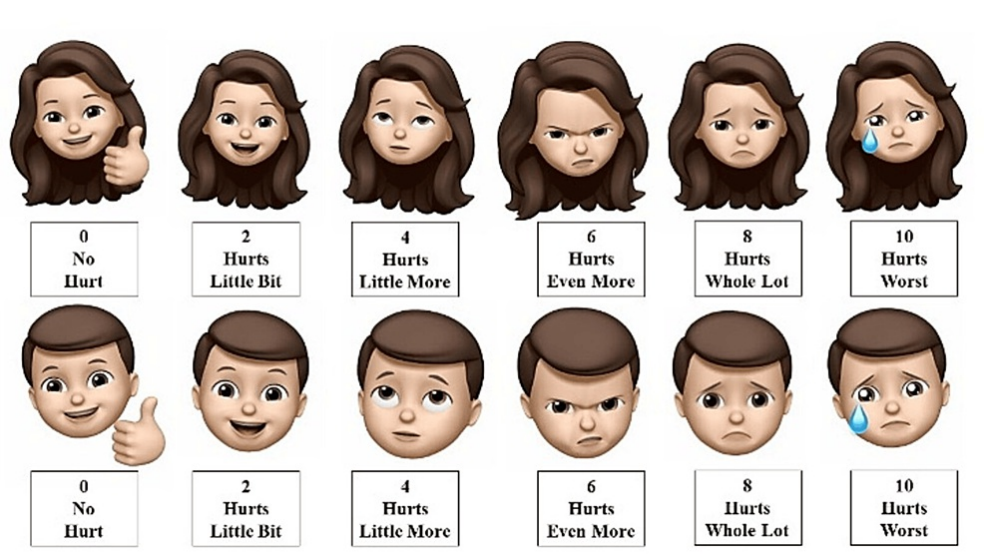
Memojis pain scale Image credit: Kanamarlapudi Venkata Saikiran

At three distinct sites, the MPS was used to assess pain five minutes after the vitality testing (Figure 3). The scale consists of six distinct Memoji characters designed to represent both boys and girls, where men are given an MPS with boy Memojis and women with girl Memojis [9]. The same investigator documented the response time and exact pain score reported by the participant in the MPS on a separate sheet of paper.

Statistical analysis

Data were entered in Microsoft Excel 2016 (Microsoft Corporation, Redmond, USA). Statistical analysis was performed using IBM SPSS Statistics for Windows, Version 17 (Released 2008; IBM Corp., Armonk, New York, United States). The Mann-Whitney U test was performed to compare the mean reaction time and mean pain scores of three sites between the upper and lower anterior teeth. The level of significance was established at 0.05.

## Results

Demographic data

A total of 180 anterior teeth (central and mandibular central incisor, lateral incisor, and canine) were included among 90 participants with a mean age of 22.86 years. A total of 540 EPT readings (three sites from 180 teeth) were made, and all tested teeth responded positively to the EPT.

Response time

The mean and standard deviation of the EPT values displayed on the device after the electrode tip placement on the upper and lower central incisors are presented in Table 1. When comparing these data, there are no statistically significant differences (p>0.05) identified among the three sites. Nevertheless, the incisal third of both the upper and lower central incisors exhibited the lowest mean value.

**Table 1:** Comparison of reaction time at various reference points among the upper and lower central incisors SD: Standard Deviation; +: Mann-Whitney U test

Type of teeth	Incisal Third Mean±SD	p-value^+^	Middle Third Mean±SD	p-value^+^	Cervical Third Mean±SD	p-value^+^
Upper Central Incisors	8.67±0.49	0.42	11.37±0.51	0.11	12.44±0.38	0.809
Lower Central Incisors	8.71±0.68		11.19±0.33		12.41±0.54	

Similarly, when the mean values of the upper and lower lateral incisors were compared, a statistically significant difference was observed between the tested sites (p<0.05). The lowest mean value was observed in the incisal third of the upper and lower lateral incisors (Table 2).

**Table 2:** Comparison of reaction time at various reference points among the upper and lower lateral incisors SD: Standard Deviation; +: Mann-Whitney U test; *:significant

Type of teeth	Incisal Third Mean±SD	p-value^+^	Middle Third Mean±SD	p-value^+^	Cervical Third Mean±SD	p-value^+^
Upper Lateral Incisors	8.64±0.21	0.000*	11.53±0.16	0.024*	12.38±0.17	0.000*
Lower Lateral Incisors	7.36±0.27		11.27±0.36		11.23±0.40	

Finally, when the mean values of the upper and lower canines were compared, a statistically significant difference was observed between the tested sites (p<0.05). The lowest mean value was observed in the incisal third of the upper and lower canines (Table 3). 

**Table 3:** Comparison of reaction time at various reference points among the upper and lower canines SD: Standard Deviation; +: Mann-Whitney U test; *:significant

Type of teeth	Incisal Third Mean±SD	p-value^+^	Middle Third Mean±SD	p-value^+^	Cervical Third Mean±SD	p-value^+^
Upper Canine	11.69±0.30	0.000*	15.40±0.26	0.03*	12.34±0.24	0.000*
Lower Canine	19.50±3.11		19.33±3.19		19.91±2.94	

Pain threshold values for upper and lower incisors (central and lateral) and canines

Table 4 represents the mean pain scores obtained from the MPS in the upper and lower central incisors. A statistically significant difference (p<0.05) among the pain scores was observed only in the incisal and cervical third, while lower mean pain scores were obtained in the cervical and middle third for the upper central incisors and the incisal and middle third for the lower central incisors.

**Table 4:** Comparison of the Memojis Pain Scale among the upper and lower central incisors SD: Standard Deviation; +: Mann-Whitney U test; *:significant

Type of teeth	Incisal Third Mean±SD	p-value^+^	Middle Third Mean±SD	p-value^+^	Cervical Third Mean±SD	p-value^+^
Upper Central Incisors	1.00±1.03	0.015*	0.00±0.00	1.000	0.00±0.00	0.000*
Lower Central Incisors	0.00±0.00		0.00±0.00		2.00±0.00	

Table 5 shows the mean MPS pain scores for the upper and lower lateral incisors. Only the incisal third showed a statistically significant difference (p<0.05) between the pain scores. A lower mean pain score was observed in the incisal third of the lower lateral incisors. All three sites exhibited the same mean pain values for the upper lateral incisors.

**Table 5:** Comparison of the memojis pain scale among the upper and lower lateral incisors SD: Standard Deviation; +: Mann-Whitney U test; *:significant

Type of teeth	Incisal Third Mean±SD	p-value^+^	Middle Third Mean±SD	p-value^+^	Cervical Third Mean±SD	p-value^+^
Upper Lateral Incisors	1.00±1.03	0.017*	1.00±1.03	0.892	1.00±1.03	0.892
Lower Lateral Incisors	0.00±0.00		1.06±1.03		0.93±1.03	

Table 6 represents the mean pain scores obtained from the MPS between the upper and lower canines. A significant difference was observed between the pain scores between the three tested sites (p<0.05). Meanwhile, lower mean pain scores were obtained in the cervical, middle third for the upper canines, and incisal third for the lower canines.

**Table 6:** Comparison of the memojis pain scale among the upper and lower canines SD: Standard Deviation; +: Mann-Whitney U test; *:significant

Type of teeth	Incisal Third Mean±SD	p-value^+^	Middle Third Mean±SD	p-value^+^	Cervical Third Mean±SD	p-value^+^
Upper Canine	1.00±1.03	0.024*	0.00±0.00	0.001*	0.00±0.00	0.001*
Lower Canine	0.25±0.68		1.00±1.03		2.00±2.06	

## Discussion

Electric pulp testing is a highly reliable and commonly used method in clinical practice to distinguish between healthy vital teeth and nonvital teeth or teeth with diseased pulp tissues. It involves applying a stimulus to the outer surface of the tooth to assess the condition of the nerves that accompany the dentin-pulp complex [10]. The EPT aims to control the health of teeth by changing ions in the neural pathway and triggering a potential response in myelinated nerves at the minimal sensory response threshold. This response is generated by delivering an electric current from the surface of the probe tip to the tooth [11]. The ideal application of the EPT probe tip to the outer surface is one of the critical factors in assessing pulp vitality [12,13]. The direction of the dentin tubules, the thickness of the enamel, the number of nerve fibers present within the underlying pulp, the size of the pulp chamber, and neural concentrations will determine the various possible sites for the placement of the probe within the same tooth [13,14]. In the present study, there was an increase in the reaction time. However, when the testing probe was moved apically, i.e. toward the tooth's cervical reference, the least reaction time was illustrated in the incisal area of the tooth. These findings can be supported because most nerve endings were located in the pulp-dentin complex near the pulp horns. The reverse, i.e., a smaller number of nerves toward the cervical area of the tooth, is also partially due to the curvature of the dentin tubules at the cervical area [15,16]. In the third incisal area, the straight path of the dentinal tubules between the enamel and dentin allows for a fast flow of current. This shortens the spatial separation between the tip and the pulp inside the tooth [17]. Therefore, it requires less time for the reaction in the incisal area compared to the middle third and cervical third, which is proportional to the minimum threshold level. These results are in congruence with various previous studies [13,17,18].

The electrical flow rate will increase in areas with rich nerve innervation and a straight orientation toward the pulp. This was supported by previous histological studies, which stated that the pulp horn is innervated with multiple free nerve endings that extend toward the pulp-dentin complex [19,20]. The dental pulp innervates two types of sensory fibers: A and C, which are myelinated and unmyelinated, respectively. The A delta fibers are the most prevalent subgroup of A fibers that coexist with unmyelinated C fibers and are pretty responsive to most of the stimuli because of their low threshold value, which does not activate the C fibers, which require a higher electrical threshold value [21]. C fibers innervate the body of the dental pulp and mediate a dull, poorly localized pain triggered only by provocations that reach the pulp properly [22]. The A delta fibers mediate sharp and aching pain and may also be aggravated by hydromechanical events that include drilling and air blowing, resulting in drying [23]. The summation effect will explain the response for threshold; it states that a satisfactory number of nerve terminals should be activated by the stimulus to achieve the response, and an increased sensory response can be achieved if there is a continuous increase in stimulus [10]. Retorting to a provided stimulus is significant in areas with more neural density, which is comparatively fast and robust and requires the slightest electric current for response [24]. In the present study, the response to the stimulus is directly proportional to the time taken for the response; that is, the response to the slightest response requires a short time, which is appreciated in the incisal area.

Similarly, in the present study, we tried to assess the existence of a correlation between the reaction time for the stimulus given from the EPT and the subjective pain scores that were gauged by employing the MPS when the stimulus was applied. Among the different pain scales, MPS was used in the present study because of its importance in determining pain effectively [25]. The study participants stated that they experienced pain for the first time when the stimulus from the EPT was applied in the incisal area for both the incisors and the canine, irrespective of the arches. The pain experienced by the participants can be attributed to sufficient A delta fibers that are healthy in function and do not constitute a quantitative evaluation of the pulp tissue. Thus, from the observations of the present study, the incisal third of the tooth can be a plausible sight for a quick and reliable assessment of the vitality of the children's teeth, considering their limited period of cooperation in the dental setting. The present study has certain limitations, including the quantitative measurement of pulp health and dependence on EPT readings derived from the pulpal nerves. Further research is required to investigate the impact of the current response on the size, condition, and nerve distribution within the dental pulp across various types of teeth and locations. Furthermore, it is recommended that future research should consider the inclusion of a larger sample size in order to enhance the applicability of the results.

## Conclusions

Given the limitations of the current study, mean threshold values of EPTs for upper and lower anterior teeth (central incisor, lateral incisor, and canine) tend to increase from incisal to middle and apical third for upper and lower central incisors and upper and lower lateral incisors at three different sites tested. Likewise, the threshold values of upper and lower canines for the incisal and cervical thirds are lower than those of the middle third. The overall scores of the MPS were 0-2 despite the site. The understanding of these variations in the EPT values across different regions of the anterior teeth can assist clinicians in accurately assessing the vitality of the teeth.
